# ENHANCING DEMENTIA CLASSIFICATION FOR DIVERSE GROUPS: TRANSFORMER-BASED CONTINUOUS SCORING OF CLOCK DRAWING TEST

**DOI:** 10.1093/geroni/igae098.4254

**Published:** 2024-12-31

**Authors:** Mengyao Hu, Yi Murphey, Tian Qin, Laura Zahodne, Richard Gonzalez, Vicki Freedman

**Affiliations:** UTHealth at Houston, Houston, Texas, United States; University of Michigan-Dearborn, Dearborn, Michigan, United States; University of Michigan-Dearborn, Dearborn, Michigan, United States; University of Michigan, Ann Arbor, Michigan, United States; University of Michigan, Ann Arbor, Michigan, United States; University of Michigan, Ann Arbor, Michigan, United States

## Abstract

Alzheimer’s disease and related dementias (ADRD) significantly impact older adults’ quality of life and pose challenges to public health systems. The clock-drawing test (CDT) is a widely used dementia screening tool due to its ease of administration and effectiveness. However, manual CDT-coding in large-scale studies can be time-intensive and prone to coding errors. In this study, we developed a deep learning neural network (DLNN) system based on Vision Transformer (ViT) to automate CDT-coding using continuous scoring and investigate its value for dementia classification, compared to ordinal CDT scores. Using a nationally representative sample of older adults from the National Health and Aging Trends Study (NHATS), we trained ViT models on CDT images to generate both ordinal and continuous scores. We compared the predictive power of these scores for dementia classification and identified demographic-specific thresholds. Continuous CDT scores provided more precise thresholds for dementia classification than ordinal scores, varying by demographic characteristics. Lower thresholds were identified for Black individuals, those with lower education, and those aged 90 or older. Compared to ordinal scores, continuous scores also allowed for a more balanced sensitivity and specificity. This study demonstrates the potential of continuous CDT scores generated by DLNN models in enhancing dementia classification. By identifying demographic-specific thresholds, it offers a more inclusive and adaptive approach for dementia classification. Results of this study could lead to improved guidelines for using CDT in dementia screening, allowing more accurate dementia classification across diverse demographic groups.

